# Complete genome sequences of two avian pathogenic *Escherichia coli* strains isolated from broilers exhibiting colibacillosis in Mississippi

**DOI:** 10.1128/mra.01020-23

**Published:** 2024-04-30

**Authors:** Linan Jia, Mark A. Arick II, Chuan-Yu Hsu, Daniel G. Peterson, Jeffrey D. Evans, Kelsy Robinson, Pratima Adhikari, Li Zhang

**Affiliations:** 1 Poultry Science, Mississippi State University, Starkville, Mississippi, USA; 2 Institute for Genomics, Biocomputing and Biotechnology, Mississippi State University, Starkville, Mississippi, USA; 3 Poultry Research Unit, USDA, Agriculture Research Service, Starkville, Mississippi, USA; University of Maryland School of Medicine, Baltimore, Maryland, USA

**Keywords:** avian pathogenic *Escherichia coli*, poultry farm, whole-genome sequencing

## Abstract

We report the whole-genome sequences of *Escherichia coli* strains APEC-O2-MS1266 and APEC-O2-MS1657 isolated from the liver and heart of infected broilers in Mississippi State, US. The genomic information of these two causative strains may provide a valuable reference for comparative studies of avian pathogenic *E. coli*.

## ANNOUNCEMENT

Avian pathogenic *Escherichia coli* is the causative agent of poultry colibacillosis, a disease with high mortality (1, 2). The genomic information of highly infectious strains can help veterinarians make efficient disease control decisions. Two *E. coli* strains were isolated previously from the hepatic and cardiac lesions in broilers that had experienced colibacillosis (3).

Lesion swabs were streaked on MacConkey agar in triplicate and incubated overnight at 37°C. Pink colonies were randomly selected and sub-cultured three times with the same conditions for pure isolation. Genomic DNA for both Nanopore and Illumina sequencing was extracted from cultures grown overnight in a 37°C LB broth using a GeneJET Genomic DNA Purification Kit (Thermo Fisher Scientific, Waltham, MA, USA). The *ybbW* gene, a gene in *E. coli* core genome and have 100% inclusivity and 100% exclusivity for *E. coli*, was amplified using qPCR for *E. coli* confirmation (4). The quantity and quality of DNA were measured using Qubit fluorometer and electrophoresis with a 0.8% wt/vol agarose gel. For long-read sequencing, the genomic DNA was fragmented with g-Tube (Covaris, Woburn, MA, USA) following the manufacturer’s procedure to generate the mean fragment size of 12–15 kb. The multiplexing library pool was prepared and barcoded using a Ligation Sequencing Kit (SQK-LSK109) and the Native Barcoding Kit (SQK-NBD104), and sequenced on an R9.4 MinION flow cell using the Nanopore GridION sequencer (Oxford Nanopore Technologies, Oxford, UK). Guppy v6.3.2 (Oxford Nanopore Technologies, Oxford, UK) was used for data base-calling. The same genomic DNA was used for short-read sequencing and a 350-bp short insert DNA-Seq library for each sample was prepared by using Illumina TruSeq DNA PCR-free Sample Prep Kit and sequenced with PE150 sequencing method on a HiSeq X-Ten sequencer (Illumina, San Diego, CA, USA). The short-reads were filtered using cutadapt (v4.3) (5) to remove any read with a TruSeq adapter sequence, and the long-reads were filtered using filtlong (v0.2.1) (6) to discard any read shorter than 1 kb and the worst 10% base on kmer overlap of the filtered short-reads. Flye (v2.9) (7) was used to create a preliminary assembly using the filtered long-reads. Unicycler (v0.5.0) (8) used the Flye assembly, filtered long-reads, and filtered short-reads to create a final, complete, circular assembly for both strains. Base coverage was calculated using SAMtools (v1.15.1) (9) with the bwa (v0.7.17) (10) aligned raw short-reads and minimap2 (v2.14) (11) aligned raw long-reads. Both the assemblies have BUSCO scores above 99.3% using BUSCO (v5.5.0) (12) and the enterobacterales_odb10 database. The sequences were annotated using the Prokaryotic Genome Annotation Pipeline (PGAP, v6.6) (13) at NCBI. Multilocus sequence typing (MLST) was identified using PubMLST (14), serotyping (O-antigen and flagellin genes) was performed using SerotypeFinder (v.2.0) (15). Default parameters were used for all software unless otherwise specified. Nanopore generated 194,007 reads with an average length of 7,731 bp for MS1266, and 103,532 reads with an average length of 7,626 bp for MS1657. Illumina generated 4,994,817 and 5,631,894 pairs for MS1266 and MS1657, respectively. Table 1 shows the genome assembly and genotypic characteristics of two *E. coli* isolates.

**TABLE 1:** Summary of genome assembly and genotypic characteristics of two *E. coli* isolates

Label	Source	Serotype	ST^*a*^	Size (bp)	Avg. per-base coverage (×)		GC content (%)	Nanopore *N*_50_	CDSs (with protein)	GenBank accession no.
					Nanopore	Illumina				
APEC-O2-MS1266^*b*^	Liver	O2/O50:H5	355	4,735,863	300	304	51	8,936	4,571	CP135959
pAPEC-O2-MS1266-1^*c*^				134,102	124	139	49			CP135960
pAPEC-O2-MS1266-2^*c*^				122,100	145	217	54			CP135961
pAPEC-O2-MS1266-3^*c*^				1,552	6	8,066	52			CP135962
APEC-O2-MS1657^*b*^	Heart	O2/O50:H1	429	5,059,841	144	319	51	8,866	4,883	CP135957
pAPEC-O2-MS1657-1^*c*^				191,500	176	389	50			CP135958

^*a*^ ST: sequencing type.

^*b*^ Chromosome.

^*c*^ Plasmid.

## Data Availability

The genome sequences and raw data are available at NCBI under the BioProject PRJNA839731. The assembled genome sequences and annotations are available at GenBank under accessions CP135957-CP135962 (Table 1). The raw data are available at the SRA (Sequence Read Archive, https://www.ncbi.nlm.nih.gov/sra/) under accessions SRR26195173, SRR26195174, SRR26195175, and SRR26195176.
